# Clinicopathological features and survival of colorectal cancer patients younger than 50 years: a retrospective comparative study

**DOI:** 10.1186/s43046-019-0006-z

**Published:** 2019-11-29

**Authors:** Robabeh Ghodssi-Ghassemabadi, Ebrahim Hajizadeh, Shaghayegh Kamian, Mahmood Mahmoudi

**Affiliations:** 1 Department of Biostatistics, School of Medical Sciences, Tarbiat Modares University, Tehran, Iran; 2 Shahid Beheshti University of Medical Sciences, Imam Hossein Hospital, Tehran, Iran; 3 Department of Epidemiology and Biostatistics, School of Public Health, Tehran University of Medical Sciences, Tehran, Iran

**Keywords:** Survival, Colorectal cancer, Young patients, Carcinoembryonic antigen (CEA), Tumor stage

## Abstract

**Background:**

Colorectal cancer (CRC) is a disease of old age, but its incidence has been rising among younger population compared to older ones. Nevertheless, there is a controversy over survival of younger patients compared to the older ones. Therefore, in the current study, we investigated the clinicopathological features and survival of the younger (< 50 years) versus older (≥ 50 years) CRC patients.

**Results:**

The younger and older groups consisted of 39.4% and 60.6% of patients, respectively. Both age groups were comparable regarding the symptom presentation and duration, and pre-operative carcinoembryonic antigen (CEA). The younger patients were diagnosed with a higher proportion of poorly differentiated (14.7% vs. 8.3%; *p* < 0.001) and more advanced tumors (53.2% vs. 45.9%; *p* = 0.266). The rectum tumor site was significantly more common among the younger patients (*p* = 0.021). The overall survival (OS) (*p* = 0.278), the cancer-specific survival (CSS) (*p* = 0.233), and the disease-free survival (DFS) (*p* = 0.497) did not differ significantly between the two groups. Based on Cox regression model, elevated pre-operative CEA level (HR = 1.41; 95%CI of 1.01–1.97), advanced tumor stage (6.06; 95%CI of 3.03–12.15), and poorly differentiated tumor (HR = 1.69; 95%CI of 1.05–2.71) were associated with decreased survival.

**Conclusions:**

The younger patients did not have poor prognosis compared to the older ones despite having an advanced tumor stage and a poor tumor differentiation.

## Background

Cancer is one of the leading causes of death and serious barrier against increasing life expectancy, worldwide. Colorectal cancer (CRC) is the fourth most prevalent cancer, contributing 9.2% of the global cancer deaths. CRC is the second leading cause of cancer death in the world [1].

In recent decades, the incidence of colorectal cancer has increased in the world [2]. About 60% of colorectal cancer cases are diagnosed in developed countries. However, CRC incidence rate has been rising in developing countries [3, 4]. Also, Iran has been experiencing an increase in the number of colorectal cancer cases [5, 6]. GLOBOCAN 2012 reported that the incidence of CRC will double among the Iranian population by 2030 [2].

Colorectal cancer is mainly a disease of the elderly. Approximately 90% of CRC cases are observed among individuals aged 55 years and older, and 50% of the cases occur in patients over the age of 75 [7, 8]. However, the incidence of CRC is increasing among young individuals in the Middle East and other regions in the world [9, 10]. The incidence of CRC among younger population in Iran is noticeably higher than that in European countries [6]. Additionally, compared to developed countries, CRC was more frequently reported among Iranians at a younger age (10 years younger) [11]. The advanced stages and poor histologic features of CRC are more likely to be observed in younger patients than in the older ones, which require more aggressive treatments [12, 13].

There is a controversy over the effects of age on the survival of CRC patients. Despite having more advanced stages of CRC in the younger group, some studies reported that the prognosis and survival of this age group are comparable to the older one, while other studies have suggested a poorer prognosis in the younger group [13–16]. The prognosis of the younger CRC patients is an important issue due to the impact of the disease and its treatment strategies on their fertility, career, life expectancy, and emotional behavior [17], which is yet unknown among CRC patients in Iran.

The aim of this study was to investigate any disparities between the age groups with respect to the survival outcomes and clinicopathological and demographic features of CRC patients.

## Patients and methods

### Patients

This is a retrospective study, in which we reviewed the medical records and pathological reports of the newly diagnosed colorectal cancer patients admitted to the Clinical Oncology Department at Imam-Hossein Hospital, Tehran, Iran, from 2008 to 2013. All newly diagnosed CRC patients younger than 75 years old were entered into the study. Patients with incomplete documents and a previous history of cancer were excluded from the study. During the study period, 439 documents meeting the age criterion were reviewed and 396 cases were included in this study, 43 cases were excluded for incomplete follow-up or incorrect contact number or incomplete document record.

### Follow-up

Patients’ routine follow-up was carried out every 3 months for 2 years, 6 months for 5 years, and thereafter every 1 year. The patients were followed up by telephone. The deadline for phone calls was May 2018. Patients’ cause of death and death time was either extracted from their medical records or asked from their first-order relatives by telephone. Patients were considered as censored at their last follow-up or the date of telephone contact if they were alive. Survival outcomes were defined as follows: cancer-specific survival (CSS), time from the date of pathological diagnosis to the date of death from CRC; overall survival (OS), time from the date of pathological diagnosis to the date of death from all causes; disease-free survival (DFS), time from the date of pathological diagnosis to the date of diagnosis of local recurrence, distant metastases, or death [18]. A patient’s survival time was calculated in months.

### Study variables

Demographic and clinicopathologic data of patients were extracted from their medical records and pathological reports. Two independent medical students were trained to review patients’ medical records and write them in a checklist. The data on age, gender, smoking status, family history of cancer diseases, body mass index (BMI), tumor stage and differentiation, tumor location, disease symptom and presentation and its starting time, pre-operative carcinoembryonic antigen (CEA), and comorbidities including hypertension, diabetes mellitus, and coronary heart disease were recorded for all patients. A CEA level equal or greater than 5 ng/ml was considered abnormal. Pre-operative CEA data was available in 187 patients. The date of local recurrence, distant metastases, or death of patients were extracted. Tumors were staged according to the American Joint Committee on Cancer (AJCC), TNM staging system, eighth edition [19].

### Statistical analysis

Continuous data are represented as mean and standard deviation or median and interquartile range (IQR). Categorical data are represented as frequency and percentage. Subjects were categorized into two groups, the younger group (age < 50 years) and the older group (age ≥50 years). Mann–Whitney and Chi-squared tests were used to compare the variables between the age groups. Kendall-tau b was calculated where necessary. A multiple imputation (MI) with five imputations was used to impute the large degree of missing pre-operative CEA data (53%). CEA level was imputed using logistic regression, and the hazard of death was included in the imputation model in addition to other study variables. Survival curves were plotted using the Kaplan–Meier method. Univariate analysis of factors thought to influence survival was carried out using log-rank test, then variables with *p* value < 0.2 were considered for proportional hazard Cox regression model. Proportional hazard assumption was assessed for the included variables in the model according to the correlation between the scaled Schoenfeld residuals with time. Statistical analyses were performed using R 3.2.1 statistical software. The “mice” package in R was used for MI. The level of significance was set at 0.05.

## Results

### Demographic and clinicopathological characteristics

In this study, there were 396 newly diagnosed CRC patients (184 women and 212 men) from March 2008 to March 2013 who were followed up until May 2018. The mean age of patients at diagnosis was 52.87 ± 11.93 years (ranged 16–75 years). Of total, 156 (39.4%) patients were < 50 years (the younger group) and the remaining 240 (60.6%) patients were > 50 years (the older group) with males totaling 84 (53.8%) and 128 (53.3%), respectively. There was no statistically significant difference between the groups with respect to gender (*p* = 0.920).

The demographic, clinical, and tumor characteristics of patients according to the age categories are represented in Table 1. Rectal bleeding was the most prevalent symptom in both age groups (47.4% in the younger group and 46.7% in the older group), followed by change in bowel habits (19.9% in younger group and 19.6% in older group). Symptom presentation and duration were similar between the two groups (*p* = 0.130 and *p* = 0.102, respectively). Both groups did not differ significantly with respect to pre-operative CEA (*p* = 0.292), BMI (*p* = 0.928), stage at diagnosis (*p* = 0.266), and positive family history of any cancer (*p* = 0.930). A higher percentage of younger patients showed CRC family history in comparison to the other group, but was not statistically significant (9% vs. 6.3%; *p* = 0.057). The younger patients had a significantly poor tumor grade than the older ones (*p* < 0.001) and comorbidities were more prevalent among the older patients (5.8% vs. 26.7%; *p* < 0.001). Rectal cancer was more common among younger patients than among the older ones (66.7% vs. 55%; *p* = 0.021).

**Table 1:** Patients demographic and clinicopathological characteristics based on age group

Variable	Category	Total (*n* = 396)	Age group		*p* value
			≤ 50 years (*n* = 156)	> 50 years (*n* = 240)	
Sex	Female	184 (46.5)	72 (46.2)	112 (46.7)	0.920
	Male	212 (53.5)	84 (53.8)	128 (53.3)	
Symptom	Rectal bleeding	186 (47)	74 (47.4)	112 (46.7)	0.130
	Change in bowel habit	78 (19.7)	31 (19.9)	47 (19.6)	
	Abdominal pain	65 (16.4)	24 (15.4)	41 (17.1)	
	Melena	38 (9.6)	10 (6.4)	28 (11.7)	
	Obstruction	16 (4)	8 (5.1)	8 (3.3)	
	Others	13 (3.3)	9 (5.8)	4 (1.7)	
BMI	< 25	197 (49.7)	78 (50)	119 (49.6)	0.928
	25–29.9	113 (28.5)	43 (27.6)	70 (29.2)	
	> 29.9	86 (21.7)	35 (22.4)	51 (21.3)	
Family history of cancer	Positive	45 (11.4)	18 (11.5)	27 (11.3)	0.930
Relativeness	First order	37 (9.3)	16 (10.3)	21 (8.8)	0.919
	Second order	7 (1.8)	3 (1.9)	4 (1.7)	
Family history of CRC	CRC	29 (7.3)	14 (9)	15 (6.3)	0.371
	Others	15 (3.8)	4 (2.6)	11 (4.6)	
Tumor site	Colon	160 (40.4)	52 (33.3)	108 (45)	0.021
	Rectum	236 (59.6)	104 (66.7)	132 (55)	
TNM stage	I	42 (10.6)	18 (11.5)	24 (10)	0.266
	II	161 (40.7)	55 (35.3)	106 (44.2)	
	III	140 (35.4)	63 (40.4)	77 (32.1)	
	IV	53 (13.4)	20 (12.8)	33 (13.8)	
Tumor grade	Well	187 (47.2)	56 (35.9)	131 (54.6)	< 0.001
	Moderate	166 (41.9)	77 (49.4)	89 (37.1)	
	Poor	43 (10.9)	23 (14.7)	20 (8.3)	
Smoker		28 (41.9)	7 (4.5)	21 (8.8)	0.106
Hypertension		37 (9.3)	4 (2.6)	33 (13.8)	< 0.001
Diabetes mellitus		26 (6.6)	3 (1.9)	23 (9.6)	0.003
Coronary heart disease		22 (5.6)	2 (1.3)	20 (22)	0.003
Other diseases		12 (3)	2 (1.3)	10 (4.2)	0.102
CEA**	< 5	271 (68.4)	102 (65.4)	169 (70.4)	0.292
	≥ 5	125 (31.6)	54 (34.6)	71 (26.9)	
Symptom duration		6 (4–12)	6 (4–12)	6 (3.6–10)	0.102

Data are presented as frequency (percent) or median (IQR) wherever it was needed

*TNM* tumor node metastasis, *BMI* body mass index, *CEA* carcinoembryonic antigen, **the imputed data are represented

### Survival outcomes and its related factors

**Fig. 1:**
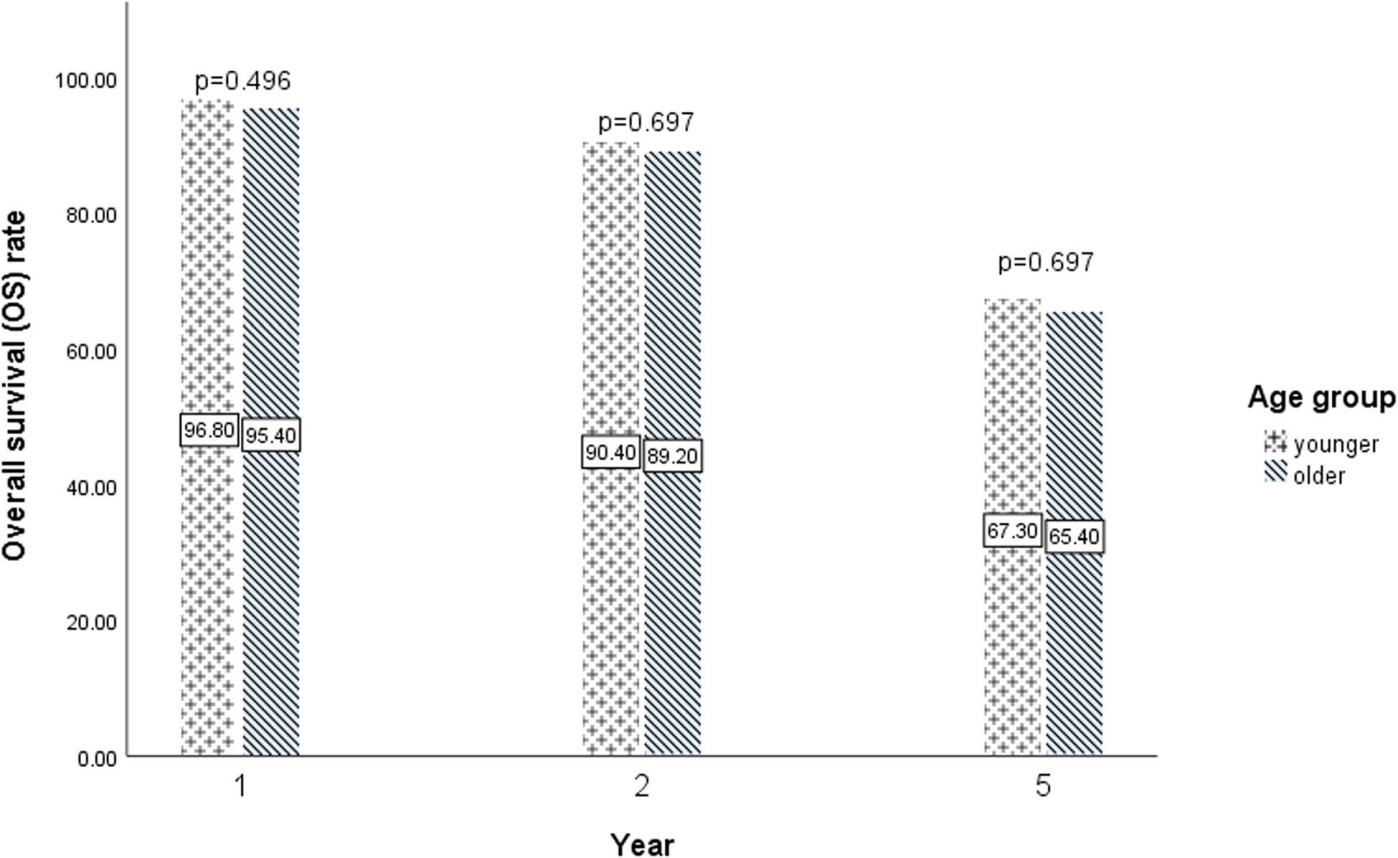
The 1-, 2-, and 5-year survival rate of patients according to the age group

The median OS, CSS, and DFS of all patients were 121 (95%CI, 97.10–144.90), 121.11 (95%CI, 97.26–144.74), and 95.80 months (95%CI, 77.955–113.65), respectively. The recurrence was observed in 48 (30.8%) and 84 (35%) younger and older patients, respectively (*p* = 0.383). The 1-, 2-, and 5-year OS rate of the patients were 96.8%, 90.4%, and 67.3% in the younger group and 95.4%, 89.2%, and 65.4% in the older group, respectively (Fig. 1; all *p* values > 0.05). Also, the 1-, 2-, and 5-year CSS rate of the patients were 97.4%, 90.8%, and 68.4% in the younger group and 95.7%, 89.3%, and 66.7% in the older group, respectively (all *p* values > 0.05). Furthermore, the 1-, 2-, and 5-year DFS rate of CRC patients were 89.1%, 77.6%, and 58.3% in the younger group and 88.8%, 77.9%, and 53.8% in the older group, respectively (all *p* values > 0.05).

**Fig. 2:**
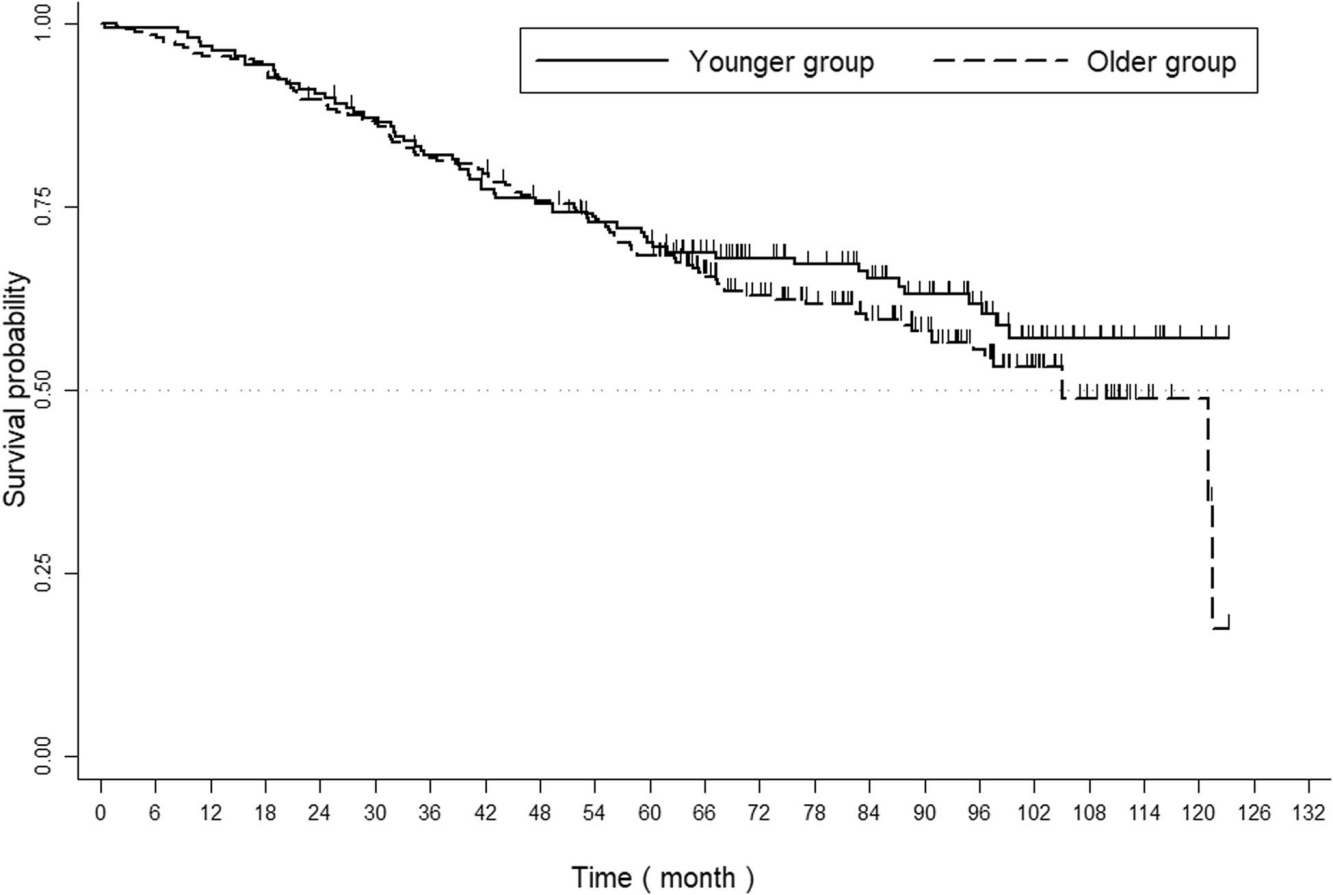
Kaplan–Meier survival curve of age groups

**Fig. 3:**
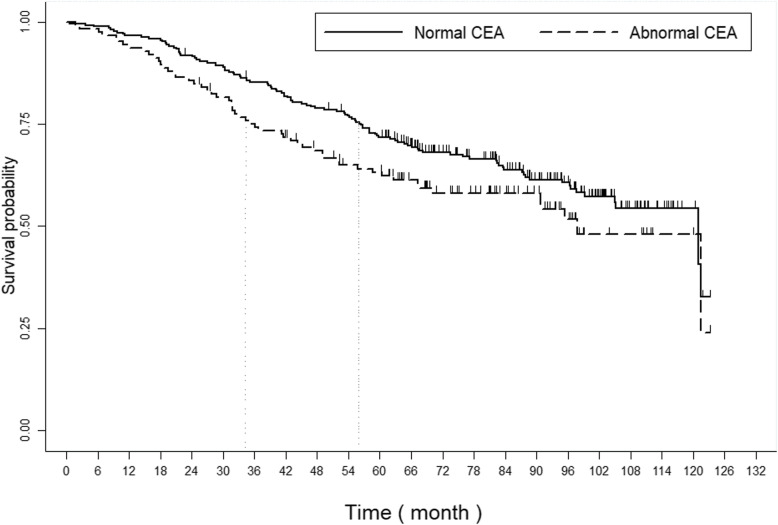
Kaplan–Meier survival curve of CEA level

For further analysis, OS was considered as the main response. Based on the results of univariate survival analysis, smoking (*p* = 0.044), advanced stages (*p* = 0.022 for the comparison of the stage III vs. I, *p* < 0.001 for the comparison of the stage IV vs. I), and poor grade (*p* = 0.002) were the only factors that influenced the survival of patients, as progress in the stage and grade of the disease decreased the survival of patients (Table 2). Figure 2 shows the Kaplan–Meier survival curve of patients with regard to the age group. The survival of patients did not differ significantly according to the age group (*p* = 0.278) (Table 2). Also, 75% of patients with normal pre-operative CEA survived more than 56 months while this period in 75% of patients with abnormal normal pre-operative CEA was 36 months (Fig. 3) and 75% of patients with stages I and II CRC survived beyond 97 and 82 months, respectively. Moreover, 75% of non-smoker patients survived beyond 52 months. By fitting the Cox regression model, it was noted that the pre-operative CEA, tumor stage, and differentiation became the only factors associated with death hazard. The elevated pre-operative CEA level (CEA ≥ 5) increased the hazard of death by 41% (HR = 1.41, 95%CI 1.01–1.97).There was a sixfold increase in the death hazard among stage IV patients compared to stage I patients (HR = 6.06, 95%CI 3.03–12.15). In addition, stage III patients had a poor survival in comparison to the stage I patients (HR = 2.29, 95%CI 1.19–4.38). Poorly differentiated histologic grade was negatively associated with the survival compared to the well-differentiated one (HR = 1.69, 95%CI 1.05–2.71) (Table 2). Furthermore, when considering the effect of pre-operative CEA level on survival based on the age group, a statistically significant decrease was observed in the survival of patients with CEA ≥ 5 in the older group (*p* = 0.019), while there was not such an association among the younger patients (*p* = 0.934) (data not shown). In complete-case analysis, regardless of the unbiased estimate of the pre-operative CEA, we did not observe any statistically significant association between pre-operative CEA and survival of patients (*p* = 0.403).

**Table 2:** Univariate and Cox proportional hazards model of patient and clinicopathological factors influencing overall survival

Variable	Category	Unadjusted HR (95%CI)	*p* value*	Adjusted HR (95%CI)	*p* value‡
Age	<50	–		–	
	≥ 50	1.19 (0.867–1.64)	0.278	1.30 (0.930–1.81)	0.125
Sex	Female	–		–	
	Male	1.35 (0.938–1.84	0.064	1.15 (0.825–1.61)	0.407
Symptom	Rectal bleeding	–			
	Change in bowel habit	1.31 (0.864–1.98)	0.204		
	Abdominal pain	1.30 (0.837–2.02)	0.243		
	Melena	1.28 (0.779–2.10)	0.331		
	Obstruction	1.14 (0.495–2.63)	0.758		
	Others	0.798 (0.291–2.19)	0.661		
BMI	< 25	–		–	0.576^b^
	25–29.9	0.728 (0.503–1.05)	0.091	0.814 (0.555–1.20)	0.294
	> 29.9	0.713 (0.472–1.08)	0.108	0.932 (0.603–1.44)	0.751
TNM stage	I	–		–	< 0.001^b^
	II	1.14 (0.590–2.20)	0.696	1.17 (0.603–2.28)	0.638
	III	2.12 (1.12–4.03)	0.022	2.29 (1.19–4.38)	0.013
	IV	6.14 (3.15–11.96)	< 0.001	6.06 (3.03–12.15)	< 0.001
Family history	Negative	–	0.319		
	Positive	0.947 (0.580–1.55)	0.829	–	–
Familial cancer	Non	–		–	
	CRC	1.05 (0.594–1.86)	0.867		
	Others	0.649 (0.240–1.76)	0.395		
Tumor site	Colon	–			
	Rectum	1.03 (0.748–1.41)	0.874		
Tumor grade	Well	–		–	0.097^b^
	Moderate	1.10 (0.790–1.54)	0.562	1.13 (0.796–1.61)	0.490
	Poor	2.06 (1.30–3.25)	0.002	1.69 (1.05–2.71)	0.031
Smoker	No	–			
	Yes	1.68 (1.01–2.77)	0.044	1.00 (0.577–1.75)	0.991
Comorbidity	No	–			
	Yes	1.19 (0.812–1.73)	0.380		
Imputed pre-operative CEA	< 5	–		–	
	≥ 5	1.34 (0.967–1.86)	0.078	1.41 (1.01–1.97)	0.046
Complete-case pre-operative CEA	< 5	–			
	≥ 5	1.52 (0.920–2.52)	0.102	1.26 (0.733–2.17)	0.403
Symptom duration		0.981 0.955–1.01)	0.160	0.984 (0.958–1.01)	0.223

*CEA* carcinoembryonic antigen, *HR* hazard ratio, *BMI* body mass index

*p value based on log-rank test

‡*p* value based on Cox regression model

^b^Overall *p* value

## Discussion

In this study, we investigated the clinicopathological characteristics and survival outcomes of CRC patients with at least 5 years of follow-up after CRC diagnosis according to the age group. Based on the findings, the median overall survival and cancer-specific and disease-free survival of CRC patients were 121, 121.11, and 95 months, respectively. The age group did not affect the prognosis of CRC patients.

Tumor stage and differentiation were important negative factors that affected the patients’ survival, as patients with stage IV disease had a poor prognosis compared to stage I patients. Furthermore, a poorly differentiated tumor was associated with decreased survival. The pre-operative CEA was a good prognostic of survival, especially among the older patients. The proportion of patients with stage IV disease was higher among younger patients. The younger patients had more advanced stages of the disease especially IV. Poorly differentiated and rectum tumors were significantly more common among the younger patients compared to the older ones.

The cutoff value of age distribution is of great importance for comparing the age groups. Age diversities might cause different results due to comorbidities contributed to the CRC [20]. The age cutoffs 30, 35, 40, 45, and 50 years have been used in different studies [13, 16, 20–23]. We used an age cutoff of 50 which is the recommended age for CRC screening in general population [24, 25].

The effect of age on patients’ survival is a matter of controversy among different studies. Our result supports the findings of Aryaie et al. They conducted the research in Golestan province, Iran, and did not observe any association between age at diagnosis and CRC survival [26]. Moreover, Yeu et al. [20] did not find a statistically significant difference on the cancer-specific survival rate of CRC patients aged under and over 50 years. Similarly, de Sousa et al. [8] reported no statistically significant difference between the young and older patients with respect to overall and cancer-specific survival. Our findings do not support some previous reports on the effects of age on CRC survival [27, 28]. This contradiction may be due to the reason that those studies considered older patients with poor general health condition that resulted in their poor prognosis, whereas, we excluded patients older than 75 years of age which were more likely to have more comorbidities and deaths other than cancer. One possible explanation for the consistent survival of the two age groups in our study, despite advanced tumors among younger patients, is contributed to the fact that some older patients due to their comorbidities did not undergo surgery or adjuvant therapy. Meanwhile, the younger patients had a lower risk of postoperative complication, better tolerance to toxicities associated with chemotherapy, and less comorbidities. The younger patients may undergo extensive surgery for tumor resection due to a better health status. Although, poor grade patients are more likely to have a poor prognosis, but probably the younger patients may undergo a more aggressive treatment which could result in a better survival [16].

According to the findings, the rectum was the most common tumor site in the younger group, which is consistent with results of a population-based study conducted by Wang et al. [28]. Jones et al. [14] reported that rectal cancer was significantly more frequent among patients younger than 50 years of age compared with the older ones. Schellerer et al. [15] reported 75% of younger patients with rectosigmoid cancer and recommended rectosigmoidoscopy for younger patients with suspicious symptoms. However, our result is in disagreement with the findings of some previous studies, in which the colon tumor was significantly more prevalent among younger patients [9, 13, 20]. Meanwhile, de Sousa et al. [8] did not find a significant difference in tumor sites between the age groups.

Our findings showed that cancer stage is the predominant factor affecting survival which is in line with a study conducted in the north of Iran on CRC patients [26]. Also, we observed a significantly higher proportion of the younger group (49.2%) with stage III or IV compared to the older one. Even a poor grade was more common in the younger group which has also been reported in previous studies [13, 20]. The presence of higher stages of cancer in the younger group might be due to delayed diagnosis, while the older patients are referred to medical center for different reasons which might lead to early diagnosis in the Iranian population. Furthermore, a CRC screening program may increase the likelihood of diagnosis at lower stages among older patients in other countries [13].

CEA is a good prognostic factor for the diagnosis and surveillance of CRC [29]. It is also considered to be a prognostic factor for the recurrence among patients with stage II [30]. In our study, survival of patients significantly differ according to the pre-operative CEA level which is in line with the findings of Zhao et al. [13]. Furthermore, subgroup analysis showed that higher pre-operative CEA led to a poor prognosis in the older group, while there was not any association between the pre-operative CEA and prognosis in the younger group. According to our findings, the hazard of death among the patients with missing CEA data did not significantly differ from those with imputed data (*p* = 0.291), but the complete-case analysis decreased the power of the effect of pre-operative CEA on the survival. So, collecting and recording the CEA data in advance of the patient’s treatment and follow-up are important and informative. In our study, the symptom presentation and duration of the younger patients did not differ significantly from the older ones, which is consistent with the results of Chan et al. [23].

To our knowledge, this is the first study which reviewed and compared the clinicopathological features and survival outcomes according to the age groups among the Iranian population which was conducted in a referral university hospital. While CRC is more prevalent among older patients, the trend of CRC incidence appears to move in an opposite direction to the age. Identifying factors affecting the survival of younger CRC patients along with better treatment strategies and careful follow-up can increase their life expectancy and ease the burden of illness on patients and health system.

## Conclusion

Despite having a more advanced cancer, the younger patients did not have a poor prognosis compared to the older ones. Early-detection strategies such as screening at a younger age may improve the survival of the younger patients.

## Data Availability

The datasets used and analyzed during the current study are available from the corresponding author on reasonable request.

## Abbreviations

BMI: Body mass index
CEA: Carcinoembryonic antigen
CRC: Colorectal cancer
HR: Hazard ratio
TNM: Tumor node metastasis
